# Monitoring of Composite Structures for Re-Usable Space Applications Using FBGs: The Influence of Low Earth Orbit Conditions

**DOI:** 10.3390/s24010306

**Published:** 2024-01-04

**Authors:** Thibault Juwet, Geert Luyckx, Alfredo Lamberti, Frank Creemers, Eli Voet, Jeroen Missinne

**Affiliations:** 1 Com&Sens, 9810 Eke, Belgium; gluyckx@com-sens.eu (G.L.); evoet@com-sens.eu (E.V.); 2 Center for Microsystems Technology, Ghent University and Imec, 9000 Ghent, Belgium; jeroen.missinne@ugent.be; 3 Engie Laborelec, 1630 Linkebeek, Belgium; alfredo.lamberti@engie.com; 4 Sabca, 3560 Lummen, Belgium; frank.creemers@sabca-limburg.com

**Keywords:** CFRP, composites, compression tests, fiber Bragg gratings, inter laminar shear strength, space, structural health monitoring, tensile testing, TVac conditioning

## Abstract

Fiber Bragg grating sensors (FBGs) are promising for structural health monitoring (SHM) of composite structures in space owing to their lightweight nature, resilience to harsh environments, and immunity to electromagnetic interference. In this paper, we investigated the influence of low Earth orbit (LEO) conditions on the integrity of composite structures with embedded optical fiber sensors, specifically FBGs. The LEO conditions were simulated by subjecting carbon fiber-reinforced polymer (CFRP) coupons to 10 cycles of thermal conditioning in a vacuum (TVac). Coupons with embedded optical fibers (OFs) or capillaries were compared with reference coupons without embedded OFs or capillaries. Embedded capillaries were necessary to create in situ temperature sensors. Tensile and compression tests were performed on these coupons, and the interlaminar shear strength was determined to assess the influence of TVac conditioning on the integrity of the composite. Additionally, a visual inspection of the cross-sections was conducted. The impact on the proper functioning of the embedded FBGs was tested by comparing the reflection spectra before and after TVac conditioning and by performing tensile tests in which the strain measured using the embedded FBGs was compared with the output of reference strain sensors applied after TVac conditioning. The measured strain of the embedded FBGs showed excellent agreement with the reference sensors, and the reflection spectra did not exhibit any significant degradation. The results of the mechanical testing and visual inspection revealed no degradation of the structural integrity when comparing TVac-conditioned coupons with non-TVac-conditioned coupons of the same type. Consequently, it was concluded that TVac conditioning does not influence the functionality of the embedded FBGs or the structural integrity of the composite itself. Although in this paper FBG sensors were tested, the results can be extrapolated to other sensing techniques based on optical fibers.

## 1. Introduction

Several approaches have been tested in the past decades for monitoring composite materials, including electrical strain gauges [1], piezoelectric sensors [2,3], and Bragg grating sensors either implemented in optical fibers (OFs) [4] or in thin flexible foils [5,6]. Among these options, fiber Bragg grating (FBG) sensors have clear advantages since they are small, can be embedded, multiplexed, and are immune to electromagnetic interference. FBG sensors have been used or have been investigated in a wide range of applications, such as structural health monitoring (SHM) [7], thermal mapping [8], process validation [9], and detection of damage, cracks, or debonding [10].

SHM is particularly crucial for re-usable space structures to ensure their structural integrity. In this regard, researchers have investigated different topics such as fatigue effects [11,12], impact damage [13,14,15,16], and delamination [17], with a focus on different sensing techniques to capture the behavior of composite components throughout their complete life cycle [18,19]. Within this context, this paper explores the influence of low Earth orbit (LEO) conditions on the performance of embedded fiber FBGs for measuring the deformation and temperature of re-usable launcher components.

In [8], a relevant overview of the research on integrated FBG sensors conducted by the European Space Agency over the last two decades is provided. Several demonstrators have been built to understand the performance, complexity, and challenges of using FBG sensors instead of their conventional electrical counterparts.

In one of those demonstrators, the need for monitoring was driven by the requirement for thermal mapping for satellites (e.g., the EUROSTAR 3000 satellite). As such, the company MPB Communications [20] integrated FBGs into various engineering models of carbon fiber-reinforced polymer (CFRP) composite antennas for the Canadian space company MDA [21]. Before embedding in the engineering models, the FBGs’ response was studied using CFRP coupons (12.5 × 200 × 0.45 mm), each having one fiber embedded, containing three FBGs on which thermal cycling (−170 °C to +135 °C; 5 cycles) was performed in a N_2_ environment. The results showed that the FBGs’ response to strain measured before and after cycling was very similar, and no delamination was observed between the CFRP layers due to the difference in extension between the fibers (glass) and the epoxy resin.

Also in [8], the VEGA launcher platform was discussed, where surface-mounted FBGs were tested to transition from electrical strain gauges to optical strain gauges and, in doing so, to minimize the mass and optimize the installation time (30 min/sensor faster). The potential for FBG sensors to replace conventional strain gauges was checked on the ESC-A and ESP stages of the ARIANE5, where both FBGs and the electrical gauges were installed and tested [8]. The results showed that FBGs function on par with electrical strain gauges for temperature and strain measurements.

In addition, FBGs were considered potential temperature and stress sensors for re-entry vehicles for the SheFEX rocket by DLR [8]. Although it was stated that FBGs were integrated within the thermal protective materials, it is not clear if thermal conditioning in vacuum (TVac conditioning) tests were conducted beforehand to check the reliability of the sensors. 

The reliability of the FBGs was also checked on the Proba-2 satellite [22], where surface-mounted sensors were used for temperature measurements. Only a slight reduction of 3 to 10% in the intensity of the reflection peaks of the FBGs was found after 7 years in LEO conditions, which did not affect the sensor functionality.

Already in 1999, the Naval Research Laboratory studied different aspects of integrated FBGs within spacecraft material [23]. Tension test coupons (according to ASTM D3039, [0]_16_ lay-up P100S/EX515 unidirectional tape prepreg) and compression test coupons (according to ASTM D3410, [0]_32_ lay-up of P100S/EX515 unidirectional tape prepreg) were produced to determine the effect of the embedded OFs with FBG sensors. Coupons with and without embedded FBGs were compared and showed no difference in tensile strength. The compressive strength was also compared between coupons with and without embedded FBGs (90° direction). No difference in compressive strength was found between coupons of both types.

A significant amount of work has been carried out using FBGs to monitor the coefficient of thermal expansion (CTE) of composite materials under LEO environments. Kang et al. [24] studied the CTE of CFRP laminates (HFG-CU-125NS carbon/epoxy prepreg tape) under LEO conditions. The reliability of the FBGs was first verified through a comparative test with electrical strain gauges on an aluminium plate. The temperature was varied between −80 and 100 °C, and the strain was measured. Excellent agreement was found, with a maximum difference of 4% between the measurements of the FBGs and electrical strain gauges. Subsequently, the CFRP laminate with embedded FBGs was subjected to 1000 thermal cycles (−70 °C to +100 °C, 2 h/cycle, 10^−6^ bar, and with UV exposure). The FBGs were used for both strain and temperature measurements. For the temperature measurement, an FBG was made free of mechanical strain by sliding it into a glass capillary tube with an inner diameter of 140 µm. As such, the FBG only responded to temperature. The measurements were then used to calculate and tabulate the CTE for different temperature ranges between −70 and +100 °C. No mechanical testing was performed to check the integrity of the structure after conditioning.

Moon et al. [25] studied the change in the reflection spectrum and the Bragg wavelength shift of FBG sensors embedded into a graphite/epoxy composite material during aging cycles simulating an LEO environment (high vacuum (~10^−5^ Torr), ultraviolet (UV) radiation (<200 nm wavelength), temperature cycling (−30 °C–+100 °C), and atomic oxygen atmosphere). The LEO aging cycles weakened the intensity of the Bragg gratings closest to the surface of the composite to approximately 70% of the initial intensity due to significant penetration of atomic oxygen. However, a clear peak remained for peak tracking algorithms, and the intensity of Bragg gratings embedded deeper in the composite was not affected.

Finally, FBGs were tested for temperature measurements in space. Kim et al. [26] studied a thermal deformation measurement system under a space environment (simulated by TVac conditioning), composed of FBG sensors for strain measurement and a displacement measuring interferometer system for accurate specimen expansion data acquisition. The results show that it is possible to precisely measure the thermal deformation of a specimen or structure in space environments using FBG sensors. In [27], a temperature sensor based on a cantilever beam with surface-mounted FBGs was developed to monitor the temperature of sandwich panels of satellites without having an effect on their mechanical performance. Mechanical and thermal vacuum tests were performed to verify the space compatibility. The space conditions were simulated as a vacuum within a temperature range from −45 to +85 °C, which is typically required for internal equipment used in space.

More generally, the influence of embedded OFs on the structural strength of composites has already been studied multiple times. In [28], tensile tests were performed on coupons with and without embedded OFs. Up to three fibers were embedded without noticeable degradation of the tensile strength. The effect of the number of embedded OFs was explored in more detail in [29]. Up to nine fibres were embedded in 25 mm wide CFRP coupons parallel to the reinforcement fibres and tested according to the ASTM-D3039M standard [30]. No degradation of the mechanical properties due to the presence of the optical fibers was found. However, for a different lay-up with 28 embedded OFs in 30 mm wide coupons, the maximum load dropped by approximately 33%, indicating a limit on the density of embedded OFs without a negative impact on the mechanical strength.

In [31], OFs were embedded in the 90° direction between the different layers of a [0/45/–45/90]_s_ lay-up. Only for the embedded fibers between the 0/45 interface was a drop in tensile strength found, while for all other cases, the tensile strength was unaffected in comparison with coupons without embedded OFs. In addition to the tensile tests, three and four-point bending tests were performed. In these tests, no difference was found between coupons with OFs embedded in between the 90/90 layers and coupons without embedded OFs. A clear degradation in bending strength was observed for the coupons with embedded OFs between the 0/45 interface. In the three-point bending test, a drop in bending strength was also observed for the coupons with embedded OFs between the 45/−45 and −45/90 interfaces. In contrast, these coupons showed an increased bending strength in the four-point bending test compared to coupons without embedded OFs and with embedded OFs between the 90/90 interface. No good explanation could be found. However, the authors hint that the bending strength of these last coupons was underestimated.

Mall et al. [32] tested the compressive strength of CFRP coupons (AS4/3501-6, various lay-ups consisting of 30 plies with 40% 0° plies, 20% 90° plies, and 40% ± 45° plies) with OFs embedded parallel and perpendicular to the reinforcement fibers and always perpendicular to the loading direction. In the worst-case scenario, with embedded OFs perpendicular to the reinforcement fibers, a reduction in compressive strength of 27% was observed. However, for all coupons with OFs embedded parallel to the reinforcement fibers, no degradation of the compressive strength was found, even for relatively large OFs (240 µm diameter).

In [33], the effects of embedded OFS on mode II fracture were investigated using end-notched flexure (ENF) testing on unidirectional composite coupons and simulations. Different lay-ups were investigated where a delamination was introduced between different layers. For all configurations, two types were produced: One without and one with embedded OFs, always between the same layers of the lay-up. The main conclusion was that, despite a reduction in the maximum load before fracture for coupons with embedded OFs, in practice, the effect becomes negligible for thick composite structures.

To the best of our knowledge, we found that, in all former and current test programs on the subject of CFRP composites with embedded OFs or capillaries, no mechanical testing was carried out post-TVac conditioning. In addition, TVac conditioning is known to induce microcracking in CFRP structures [34]. Therefore, the goal of this paper was to assess the impact on the structural integrity of CFRP composites from embedded OFs in the operational phase of relaunchable space structures and thus subject them to LEO conditions. These conditions were simulated using TVac conditioning. In addition, the reliability of the embedded FBGs for strain measurements in LEO conditions was studied. The paper starts by explaining the choice of the specific space conditions that were simulated during TVac conditioning, followed by an overview of the methods and materials used. Subsequently, the results are shown and discussed.

## 2. LEO Conditions

During the lifetime of a space structure, different stages can be identified:

- MAIT (manufacturing, assembly, inspection, and testing) phase;
- storage, transportation, and handling;
- in-service phase.

For all these phases, specific requirements apply to the structure as well as to the monitoring system, in this case, the integrated optical fiber sensors (FBGs, in this work). During the MAIT and the storage, transportation, and handling phases, the conditions differ greatly compared to the LEO environment to which the structure will be exposed during the in-service phase. In a survey for the NASA Electronic Parts and Packaging (NEPP) Program [35], the LEO conditions were described as operating conditions during which the temperature cycles between −65 °C and +125 °C at a maximum pressure of 10^−10^ Torr. The number of experienced cycles per year depends on the orbit height and varies between 780 (at 20,000 km) and 6000 (at 2000 km) cycles.

These conditions are commonly simulated using TVac conditioning (explained below), a standard approach to testing and qualifying components for space applications. After careful consideration with the ESA technical officer of the “Life cycle monitoring” project (ESA contract nr.: 4000136778/21/NL/AR), it was decided to perform 10 TVac cycles. Although 10 cycles is not enough for full TVac testing of the materials, this was considered sufficient for “re-usable” launchers, which have short exposure times to LEO conditions.

## 3. Materials and Methods

The goal of this work was twofold: (i) Assessing the influence of the embedded OFs and capillary on the composite structural strength with and without TVac conditioning; and (ii) assessing the influence of the TVac conditioning on the correct functioning of the FBGs. For the CFRP material, this involved checking its structural strength in tension and compression, as well as the interlaminar shear strength, with and without TVac conditioning.

Therefore, the first step within this work was to produce CFRP coupons (56 in total) using automatic tape laying (ATL) and submit 40 of these to TVac cycling. A comparison was made between the conditioned and unconditioned coupons, as well as between coupons with and without embedded OFs or capillaries. The tests foreseen within this work were based on determining the ultimate tensile strength (UTS) and ultimate compressive strength (UCS) according to the commonly used ASTM D3039 [30] and D3410 [36] standards, respectively, and apparent interlaminar shear strength (ILSS, NBN EN 2563 standard [37]). In this work, the strain measurements were performed using FBG sensors. Therefore, its working principle is described first, followed by an overview of the manufactured coupons, the TVac conditioning process, and the visual inspection method. The section is then concluded by describing the three mechanical tests performed: tensile, compression, and ILSS testing.

### 3.1. FBG Working Principle and the Use of Capillaries

An FBG sensor consists of an optical fiber in which a Bragg grating is inscribed [38]. As a result, a specific wavelength is reflected, while all other wavelengths are transmitted further into the optical fiber. The reflected wavelength or “Bragg wavelength” is determined by the Bragg equation λ_Bragg_ = 2nΛ, where Λ is the pitch and n is the effective index.

**Figure 1:**
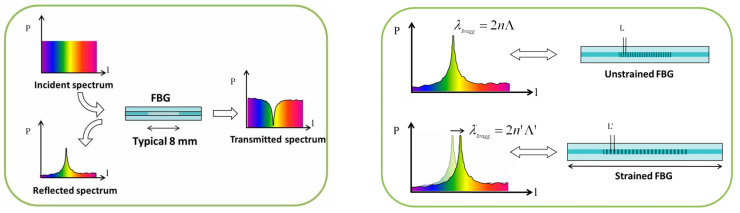
Schematic overview of the working principle of FBGs. Courtesy of FBGS [39].

Figure 1 illustrates how such an FBG sensor is used for strain sensing, i.e., when axial strain is applied to the sensor, both the grating period and the fiber refractive index change, resulting in a change in Bragg wavelength (shift to longer wavelengths for positive strain). In addition, if temperature changes, the Bragg wavelength will also change (due to thermal expansion of the grating and due to thermo-optic effects), so FBG sensors can also be used as temperature sensors. However, if the sensor is subjected to mechanical load and temperature changes at the same time, extra measures are required to distinguish the effects of both. The easiest way to achieve a strain-insensitive temperature sensor lies in the design of the package, which mechanically isolates the FBG element from strain, e.g., in the form of a capillary [8]. A Teflon, polyimide, or PEEK tube was suggested as an external probe, and different TVac cycling (−50 °C to +125 °C) tests were performed, showing the functionality of FBGs as temperature sensors under space conditions.

**Figure 2:**
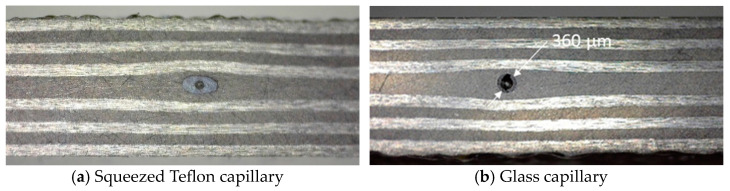
The cross-section of 2 coupons (lay-up [0/90]_3S_) with embedded Teflon (**a**) or glass (**b**) capillary.

In this paper, the same method was used to realize strain-insensitive temperature sensors. Since Teflon tubing was found to be incompatible with the ATL process used to manufacture the coupons (the tubing was squeezed, as shown in Figure 2a), a glass capillary was used (which retained its form as shown in Figure 2b). An FBG-scan 808D interrogator (bandwidth: 1510–1590 nm, resolution 0.8 nm/pixel) from FBGS (Jena, Germany) was used to acquire the FBG reflection spectra and to perform the stain measurements. The used FBGs were off-the-shelf draw tower gratings (DTG), also obtained from FBGS, which have a length of 8 mm, can measure strains up to 5% and temperatures between −200 and +200 °C, and have a typical strain sensitivity of 1.2 pm/µε and a temperature sensitivity of 10 pm/°C.

### 3.2. Coupon Geometry

**Figure 3:**
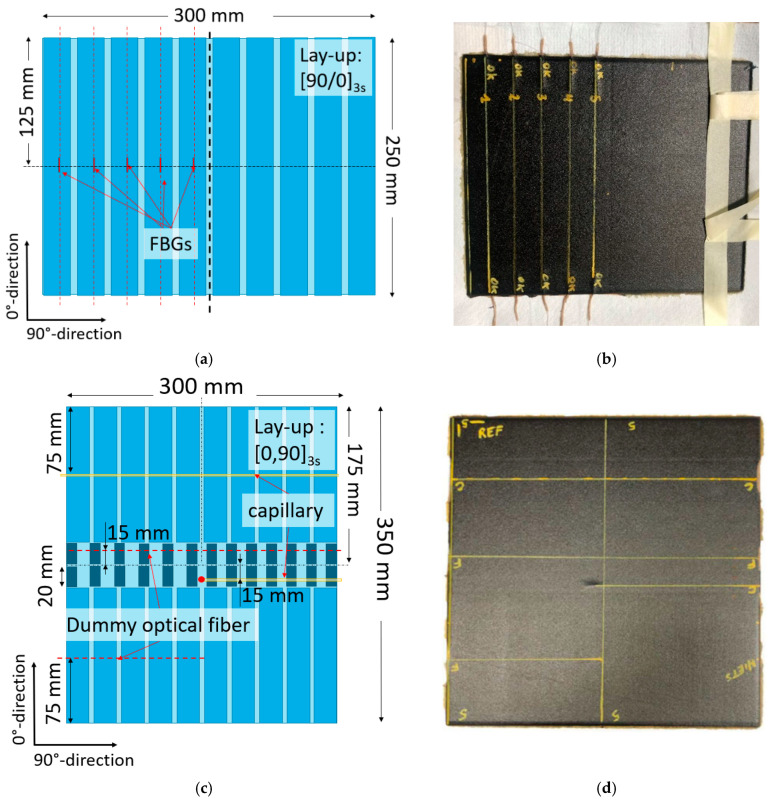
(**a**) Schematic overview of the produced test plate and how the coupons for the tensile test were cut out. The location of the embedded OFs is shown with the red dashed line. The FBG was located in the middle of the coupon between two 0° degree layers. (**b**) The produced plate just before it was cut into individual coupons for the tensile test. The yellow lines showed where the OFs with FBG sensors were embedded. (**c**) A schematic overview of the produced test plate and how the coupons for the compression and ILSS tests were cut out. Dummy OFs (red dashed line) without FBGs and glass capillaries (yellow line) were embedded at the indicated locations between two 90° layers. (**d**) The produced plate just before it was cut into coupons for compression and ILSS testing. The yellow lines indicated where the OFs or capillaries were embedded.

Two plates (Figure 3) were manufactured by stacking unidirectional prepreg layers (Hexcel HexPly_8552_34%_UD194_AS4 [40]) using the ATL process. The coupons were subsequently cut out using a diamond blade table saw, according to the dimensions stated in the ASTM D3039 [30] and D3410 [36] standards and the NBN EN 2563 standard [37] for the tensile, compression, and ILSS tests, respectively.

For the first plate, a [90/0]_3s_ lay-up was used, and 5 optical fibers (80/120 µm fiber/cladding diameter) with FBGs were embedded in the 0° direction between the 2 middle 0° layers. The FBGs were embedded parallel to the reinforcement fibers to avoid Bragg peak distortion [41]. The plate had a nominal thickness of 2.5 mm and measured 250 by 300 mm, and 10 coupons were cut out of 250 mm in length and 25 mm in width. A schematic overview with dimensions is shown in Figure 3a. A photo of the fabricated plate is shown in Figure 3b. The yellow lines indicate the locations of the embedded FBGs.

The coupons used in the compression and ILSS tests were cut out of a second plate of 300 by 350 mm with a [0/90]_3s_ lay-up and nominal thickness of 2.5 mm. Figure 3c,d shows a schematic overview of the panel layout and the final plate. The ILLS test coupons are shown in darker blue and were 20 mm long and 10 mm wide. The coupons for the compression test were 150 mm long and 25 mm in width. Dummy OFs (an 80/120 µm fiber/cladding diameter, without FBG) and glass capillaries (Polymicro glass capillary coated with polyamide) were embedded in the 90° direction between the 2 middle 90° layers. A capillary with a 250/360 µm inner/outer diameter was embedded in the middle of 10 coupons for the compression test, and a dummy OF was embedded in the middle of another 5 coupons for compression testing. As such, 5 compression test coupons were left without embedded OF or capillary for reference. For the smaller ILSS test coupons, a 106/160 µm inner/outer diameter capillary was embedded in 8 coupons. A dummy OF was embedded in 10 ILLS test coupons. The capillary and OF were embedded at ¾ of the length of the coupons. Eight reference coupons without embedded structures were manufactured.

**Figure 4:**
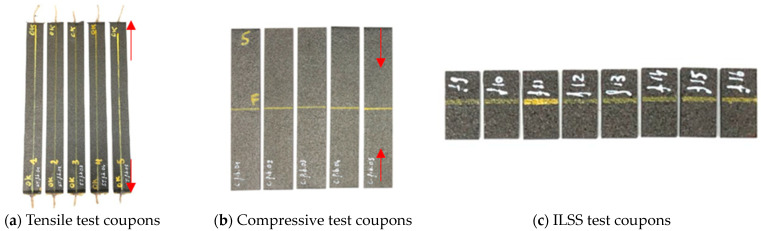
Photo showing some of the coupons used for (**a**) the tensile test, (**b**) compression test, and (**c**) ILSS test. The direction of the embedded OF/capillary is shown with the yellow line. The direction of the applied force during the test is shown by the red arrows. For the ILSS test, the force was applied out of plane.

Figure 4 shows the 3 different coupon types after fabrication: (a) The tensile test coupons, (b) the compression test coupons, and (c) the ILSS test coupons. The red arrows give the direction used for the loading conditions, and the yellow lines show where OFs or capillaries were embedded. An overview of the dimensions and quantities of the manufactured coupons is given in Table 1 and Table 2, respectively.

### 3.3. TVac Conditioning

**Figure 5:**
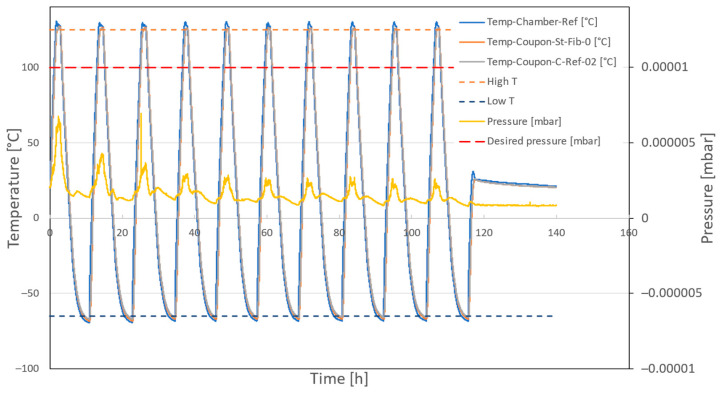
The measured temperature and pressure inside the autoclave during the TVac conditioning of the coupons. The dashed lines indicate the desired minimum and maximum temperature and maximum desired pressure. The yellow curve shows the measured pressure in the autoclave during TVac conditioning. The blue, orange, and grey curves show the temperature as measured with three thermocouples inside the autoclave. Two of these were taped to a coupon, and the third one was taped to the platform in the autoclave.

The coupons were placed in an autoclave where a vacuum was created with a maximum pressure of 10^−5^ mbar, and the temperature was varied between −65 °C and 125 °C for 10 cycles. The measured temperatures and pressure during the cycling are depicted in Figure 5. The temperature was measured with three thermocouples; two of these were taped to a coupon, and the third was taped to the platform in the autoclave.

### 3.4. Visual Inspection

To visualize the embedded OFs and capillaries, the coupons were hand polished. The polishing was performed in steps, starting from 1500 up until 3000 grit size sandpaper, and then finalized with 1 µm grit size sandpaper. The visual inspection was performed using a USB microscope camera (Dino-Lite DXL.AM.4515.ZTL, AnMo Electronics Corporation, New Taipei City, Taiwan). Pictures were taken before and/or after TVac conditioning and compared.

### 3.5. Tensile Testing

The tensile test was performed according to the ASTDM D3039 testing standard [30]. The main objective was to assess the functionality of the embedded FBGs and determine the influence of embedded OFs on the ultimate tensile strength (UTS) after TVac conditioning.

**Figure 6:**
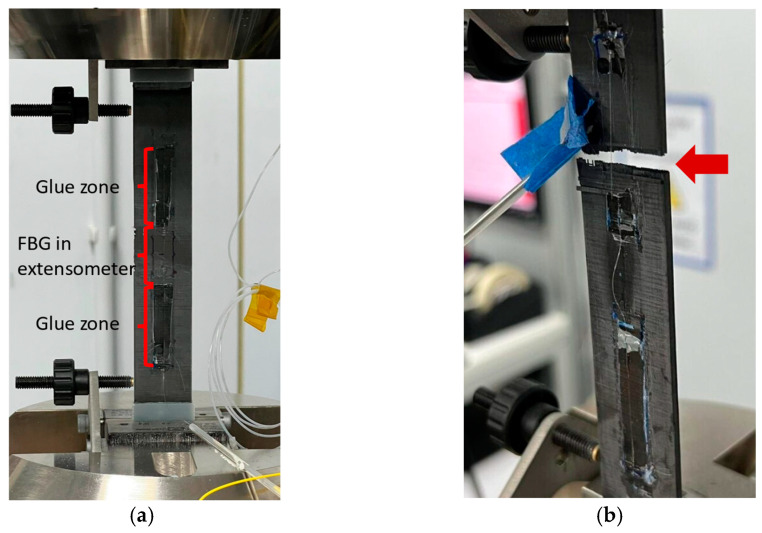
A tensile test coupon mounted in the test machine (**a**) before and (**b**) after the tensile testing. The FBG installed in extensometer principle after TVac conditioning can be seen. The optical fiber was glued to the surface of the coupon on both sides of the FBG sensor ((**a**), glue zone), leaving the FBG itself free. As such, two anchors were created, which strained the FBG sensor during the experiment. (**b**) shows the fracture after tensile testing.

Five coupons were made with embedded FBG sensors and five without. All coupons were subjected to TVac conditioning and were then equipped with tabs and a reference strain sensor (FBG used in the extensometer principle (see Figure 6a). Subsequently, the coupons were tested by applying a tensile load until a fracture occurred. The tensile load was applied in the 0° direction, along the direction of the embedded OFs (see Figure 4). Figure 6 shows an example of a coupon mounted on the test bench before and after testing. The measurements of the reference strain sensors were compared with the measurements of the embedded FBG to determine the proper functioning of the embedded FBG after TVac conditioning.

The test was carried out on an Instron 8810 servo-hydraulic testing machine (Instron, Norwood, MA, USA) with a 100 kN load capacity. The load was measured with the load cell on the test bench. Combining the measurements of the load cell and the FBG measurements, a typical stress (σ)–strain (ε) curve was obtained. The stiffness value was then calculated from the slope of this curve:

(1) $$E\;[Gpa]=\frac{\sigma \;[kpa]}{strain\;[\mu \varepsilon ]},$$

### 3.6. Compression Testing

The compression test was performed according to the ASTM D3410 testing standard [36]. The main objective was to determine the influence of embedded OF and capillaries on the ultimate compressive strength (UCS) after TVac conditioning.

For this test, 20 coupons were manufactured: 5 coupons with embedded OFs, 5 reference coupons, and 10 coupons with embedded capillaries. Except for 5 coupons with embedded capillaries, all coupons were TVac conditioned.

**Figure 7:**
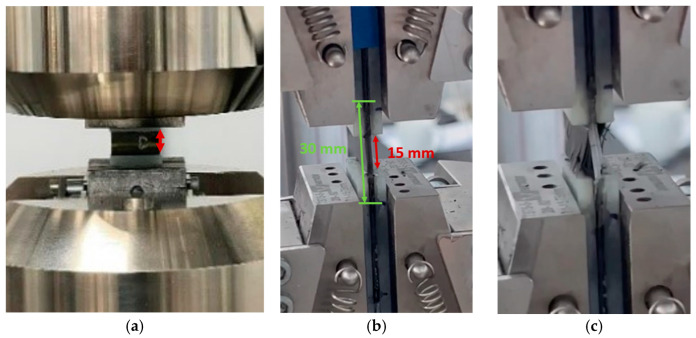
A coupon clamped in the test machine and a close-up before (**a**,**b**) and after (**c**) test. The green arrow in (**b**) shows the length over which the FBG is glued to the edge of the coupon. The FBG itself is located in the middle and is only 8 mm in length. The red arrows (**a**,**b**) show the free length of the coupon.

An example of a mounted coupon before and after testing is shown in Figure 7. The coupons were equipped with tabs, leaving 15 mm of free length (shown in red in Figure 7a,b). A reference strain sensor was applied by gluing an OF with FBG at the side of the coupon over a length of 30 mm (shown in green in Figure 7b). The coupons were clamped in and subsequently tested using a compressive load until fracture. The compression load was applied in the 0° direction, perpendicular to the direction of the embedded OF and capillary and perpendicular to the direction of the reinforcement fibers of the layers in which the OF and capillary were embedded. As such, this was the most critical case in terms of the loading direction versus the supporting reinforcement fibers’ direction. For this reason, it was chosen to embed capillaries in the compression and ILSS (see Section 3.7) test coupons and not in the tensile test coupons. 

The test was carried out on an Instron 8810 servo-hydraulic testing machine with a 100 kN load capacity. The load and displacement were measured with the load cell on the test bench. In addition, the compressive strain was measured using the FBGs mounted at the side of the test coupon. The stress–strain curve was obtained by combining the measurements of the load cell and the reference FBGs and was used to calculate the ultimate compressive stress of the material and the stiffness value (Equation (1)).

### 3.7. Inter Laminar Shear Strength (ILSS) Testing

The ILSS testing was performed according to the NBN EN 2563 testing standard [37], which is used to determine the apparent interlaminar shear strength of composite materials. In principle, this standardized test is conceived for unidirectional laminates. For cross-ply laminates, the test cannot be used to determine the absolute interlaminar shear strength. However, a comparison can be made between laminates with an identical lay-up. The main objective was the latter, to determine the influence of embedded OF and capillaries on the apparent interlaminar shear strength (ILSS) of the CFRP coupons after TVac conditioning.

For this test, 26 coupons were manufactured: 8 without embedded OFS or capillary, 10 with embedded OFS, and 8 with embedded capillary. For each category, 5 coupons were TVac conditioned. In addition, 6 more coupons were manufactured with embedded OF, of which 3 were also TVac conditioned. These 6 extra coupons were also visually inspected (see Appendix A.3). However, they were not used during the ILSS tests (and are not taken into account in Table 1 and Table 2). Due to the small dimensions of the coupons, no reference strain sensor could be mounted.

**Figure 8:**
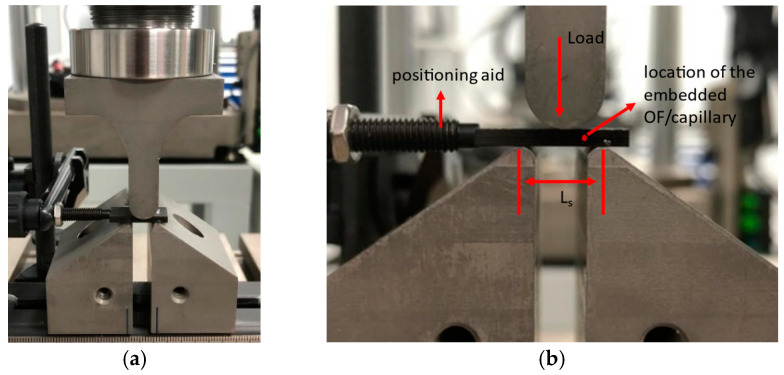
(**a**) Shows an ILSS test coupon mounted in the 3-point bending test setup. (**b**) shows a close-up of the mounted coupon. The support span L_S_ was 10 mm. The red dot indicates the position of the embedded OF or capillary, which was positioned in the middle between the support and loading point.

A coupon mounted on the three-point bending is shown in Figure 8. Figure 8a shows an overview, while Figure 8b shows a close-up of the mounted coupon. The loading span (L_s_) was 10 mm and the coupon was positioned so that the embedded OF or capillary was located halfway between the loading point and support. The test was performed until delamination occurred.

The test was carried out on an Instron 5985 electromechanical testing machine with a 10 kN load capacity. The load was measured with the load cell on the test bench, and the load at failure was recorded. The apparent interlaminar shear strength (τ) for a cross-ply laminate was then calculated as follows:

(2) $$\tau =\frac{3P_{R}}{4b\cdot h},$$

where:

$P_{R}$ is the maximum load at first ply failure;

b is the width of the coupon;

h is the thickness of the coupon;

To be valid, the coupon should break at its neutral axis, which was determined post-mortem.

## 4. Results and Discussion

### 4.1. Functionality Testing of Embedded FBGs Subjected to TVac Conditong

**Figure 9:**
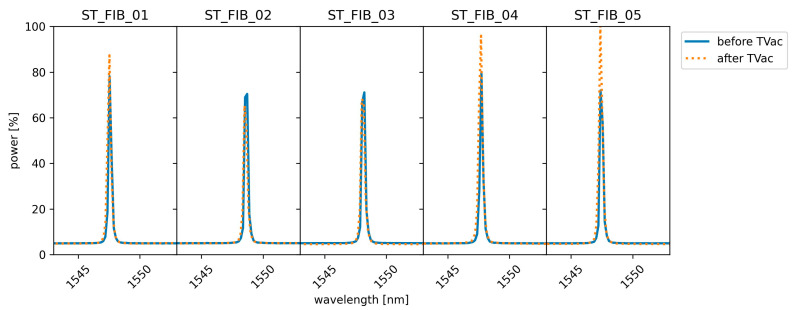
Comparison of the reflection spectra of the embedded FBGs in the 5 tensile test coupons taken before and after TVac conditioning at room temperature. No degradation of the spectra was observed.

To check the functionality of the FBGs embedded in the tensile test coupons before and after TVac conditioning, the reflection spectra were taken before and after TVac conditioning at room temperature, the results of which are shown in Figure 9 (per coupon). No peak distortion or significant reduction in reflection could be seen in the spectra after 10 cycles of TVac conditioning, meaning that peak tracking (needed for strain measurements) remained possible, ensuring the sensor’s functionality. This is in correspondence with the results found in [22] on the temperature sensors on the PROBA-II mission and in [25], where LEO conditions were simulated by TVac cycling in combination with UV radiation and an atomic oxygen environment.

In addition, strain measurements of the surface-mounted reference sensors were compared with those of the embedded FBGs during the tensile test. The ultimate strain (strain at failure) as measured by the embedded and surface-mounted sensors is given in Table 3. The mean correlation was 1.003 (±0.008), which shows the embedded FBGs capture the strain perfectly and no degradation of strain transfer from the CFRP to the embedded FBGs occurred due to TVac conditioning.

### 4.2. Mechanical Strength Testing of CFRP Coupons with Embedded OF after TVac Conditiong

#### 4.2.1. Visual Inspection

**Figure 10:**
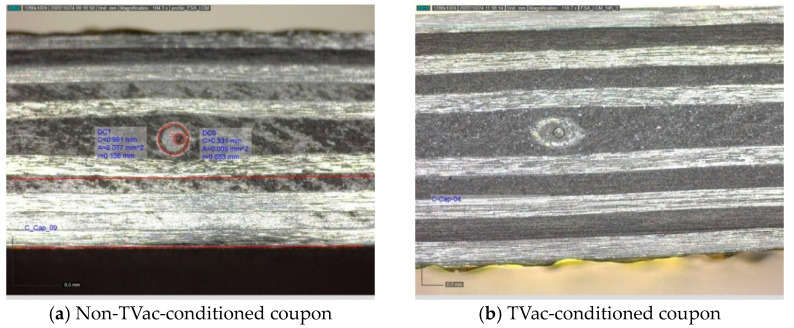
Example of 2 polished compression test coupons with an integrated 250/360 µm capillary.

**Figure 11:**
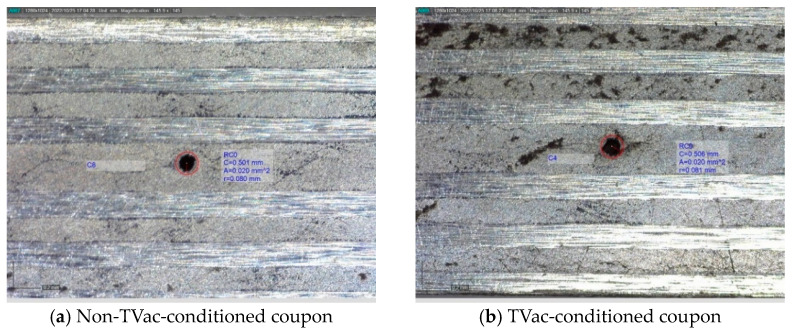
Example of 2 polished samples from the ILSS test coupons with an integrated 106/160 µm capillary.

**Figure 12:**
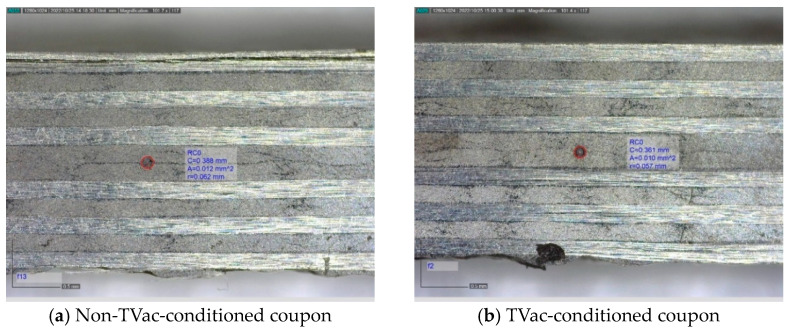
Example of 2 polished samples from the ILSS test coupons with an integrated 80/120 µm OF.

**Figure 13:**
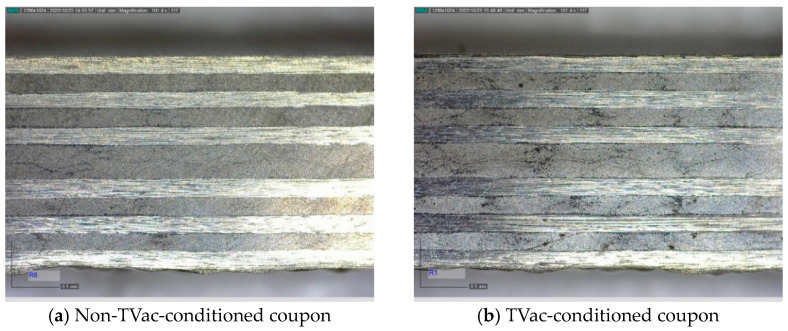
Example of 2 polished samples from the ILSS test coupons without an embedded OF or capillary.

Figure 10, Figure 11, Figure 12 and Figure 13 show examples of polished coupons with embedded 250/360 µm and 106/160 µm inner/outer diameter capillaries, embedded OFs, and coupons without embedded structures. In these figures, (a) shows the polished cross-section of a coupon that was not TVac conditioned and (b) shows another coupon that was TVac conditioned. No delamination or degradation due to TVac cycling could be seen. The same result was found for all the coupons (Appendix A). A comparable result was found in [8], where it was mentioned that no delamination was found in coupons with embedded OFs after 5 thermal cycles (−170 °C–+135 °C in an N_2_ environment).

The breakage of the capillary, as shown in Figure 10b, seems to originate from the manufacturing process, which is proven by the presence of resin within the capillary (lighter color in the image; otherwise, this should have been black like in Figure 11). In the following experiments, this has been avoided by placing the capillary in between two ATL prepreg tapes, leading to a better accommodation of the capillary within the CFRP.

#### 4.2.2. Tensile Test

**Figure 14:**
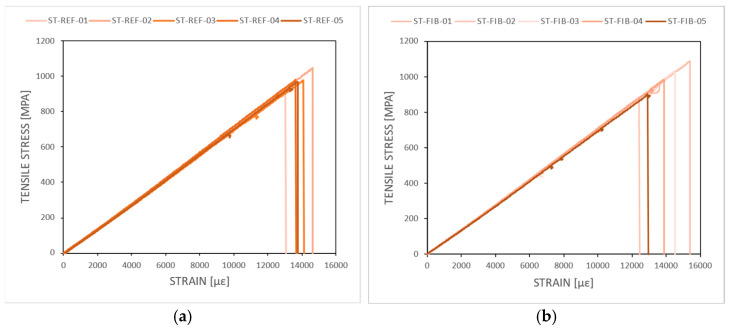
The stress–strain curve as a result of the tensile test obtained by combining the load data from the test bench with the strain measurements from the FBGs applied in extensometer principle. (**a**) Shows the results for the reference coupons (“ST-REF”, no embedded OF or capillary). (**b**) Shows the results for coupons with embedded OF (“ST-FIB”).

**Figure 15:**
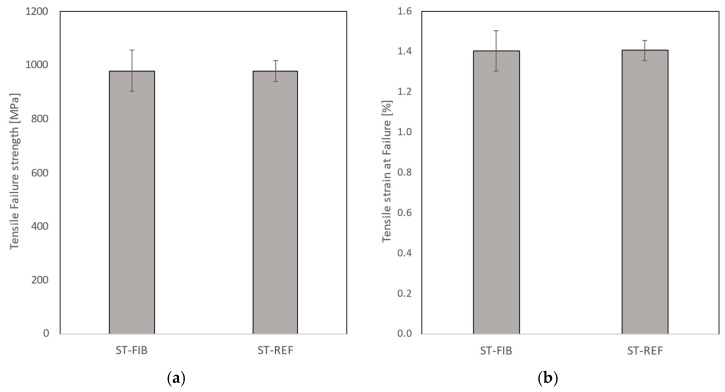
(**a**) The tensile failure strength and (**b**) tensile strain at failure obtained from the quasi-static tension testing. Coupons with (“ST-FIB”) and without (“ST-REF”) embedded OF after TVac conditioning were compared.

Figure 14 shows the stress–strain curves resulting from the quasi-static tension test for all 10 coupons (5 with embedded OF with FBG and 5 without) that were tested after being TVac conditioned. A bar chart summarizing the tensile failure strength and the tensile strain at failure (incl. standard deviations) is depicted in Figure 15, while a detailed overview of all individual test results is given in Appendix B.1.

No difference in tensile strength (Figure 15) is observed between both types, although a slightly higher standard deviation is seen for the coupons with embedded OF, caused by one coupon (ST-FIB-02) that exhibited a lower tensile strength value. Comparable tests have been performed on unconditioned coupons in [23,27,29]. Various lay-ups were tested with embedded OFs parallel to the reinforcement fibers and no reduction in tensile strength was found compared to coupons without embedded OFs when the amount of embedded OFs is limited [29]. In [31], the influence of the angle of the embedded OF with respect to the interface of the layers in which it was embedded was tested. A reduction in tensile strength was found only when the OF was embedded in the 90° direction in a 0/45 interface.

The calculated mean stiffness values were 71.26 ± 0.71 GPa and 71.58 ± 0.71 GPa for the coupons with and without embedded OF. In the technical data sheet [40], a tensile strength of 141 GPa //, and 10 Gpa ┴ in the case of unidirectional prepreg is given. However, given the [90/0]_3s_ lay-up used, this amounts to a theoretical stiffness of 75.5 Gpa in the 0°direction, which is only slightly more than the measured stiffness values, indicating that TVac conditioning did not influence the structural strength of the coupons significantly, either with or without embedded OF.

#### 4.2.3. Compression Test

**Figure 16:**
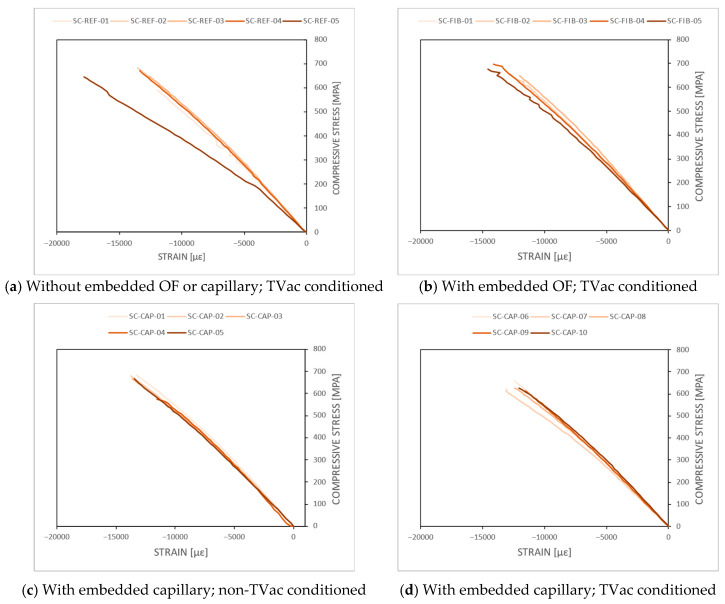
The stress–strain curves resulting from the quasi-static compression tests for the compressive test coupons. The stress was measured by the load cell, and the strain was obtained with the FBG sensors.

**Figure 17:**
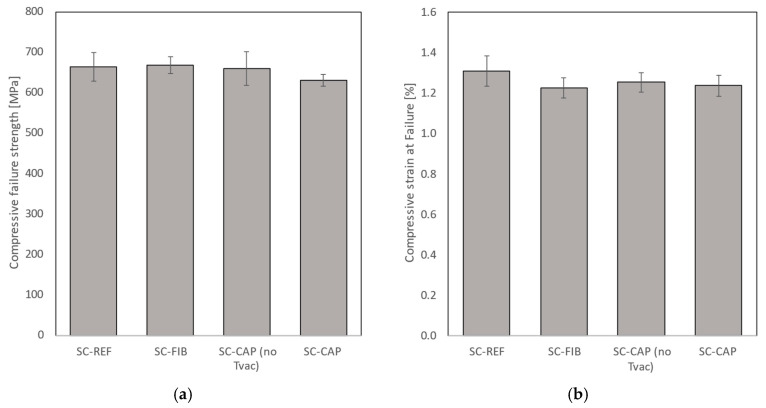
(**a**) Compressive failure strength and (**b**) compressive strain at failure obtained from quasi-static compression testing. TVac-conditioned coupons without embedded structures and with embedded OFs and capillaries are compared as well as coupons with embedded capillaries but which were not TVac conditioned.

The stress–strain curves resulting from the quasi-static compression tests, combining the FBG strain measurements and the stress measurements (load divided by cross-sectional area) from the test bench, are shown in Figure 16 for all 20 coupons. In Figure 16a, it can be seen that the strain measurement of coupon SC-REF-05 showed high strain values in comparison to the other SC-REF-0X coupons, which should be interpreted with care and was ignored for further data analysis (see also Appendix B.2). High spectral distortion was seen by the FBG sensor of SC-FIB-01 and was therefore also ignored. An overview bar chart of the measured compressive strain at failure and compressive failure strength is depicted in Figure 17, including the standard deviation, and an overview of all individual results is given in Appendix B.2.

No significant difference in compressive strength was observed among the different coupon types. The mean compressive strength of the TVac-conditioned coupons with embedded capillaries was 631.24 ± 16.22 MPa, which is slightly lower compared to the mean compressive strength value of their unconditioned counterparts, 660.46 ± 46.57 MPa. However, this difference falls within the standard deviation of the latter, indicating no significant variation in compressive strength between unconditioned and conditioned coupons.

As the free length of the coupons clamped in on the test bench is shorter than the gluing length of the reference FBG glued on the side, the strain measured in this test is not reliable to determine the absolute strain at failure, as incomplete strain transfer from the coupon to the FBG could occur. However, these measurements can still be utilized to compare the different coupon types (REF, FIB, and CAP). The results indicate that, although small changes were observed between the different types of coupons, no significant effect related to the TVac conditioning could be discerned.

The stiffness values were calculated from the stress–strain curves, yielding mean values of 57.4 ± 1.2 GPa, 56.3 ± 2.7 GPa, 57.4 ± 2.1 Gpa, and 57.9 ± 2.1 GPa for the REF, FIB, CAP (no TVac), and CAP coupons, respectively. As the strain values are not absolute material properties, this applies equally to the stiffness values. However, the results once again demonstrated that no discernible differences were found between TVac-conditioned and non-TVac-conditioned coupons and among different types. This finding is consistent with earlier studies [23,32] using unconditioned coupons.

#### 4.2.4. ILLS Test

**Figure 18:**
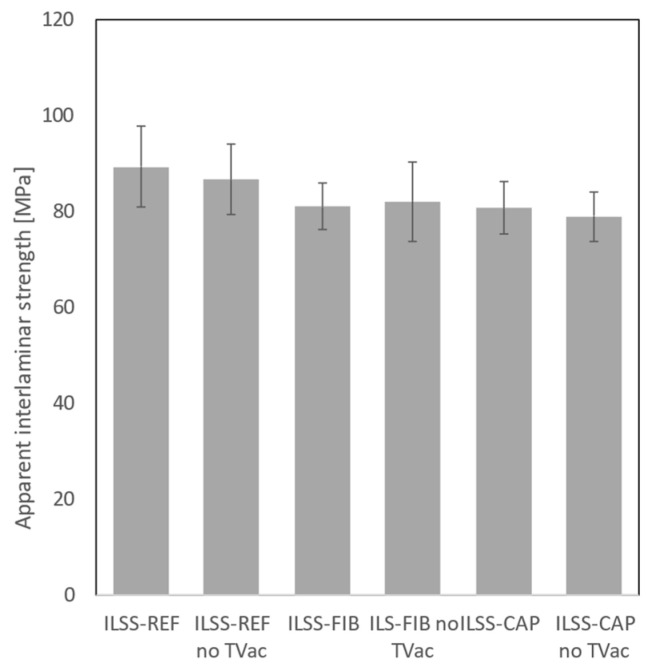
Bar chart of the apparent interlaminar shear strength for the different types of coupons.

Figure 18 shows the mean apparent interlaminar strength of the coupons. An overview of all results is given in Appendix B.3.

A slight decrease in interlaminar shear strength of about 8% was observed for the coupons with embedded OF (“FIB”) or capillary (“CAP”) compared to the reference coupons (“REF”). Given the small size of the coupons, it was expected that the effect of embedding a non-carbon fiber or capillary would be more pronounced due to a decrease in the volume fraction of the carbon reinforcement fiber. Previous research [32,33] reported similar results for tests conducted on unconditioned coupons. It was concluded that the influence of embedded optical fibers is minimal, if not negligible, given some specific restrictions. These restrictions can in principle be summarised as follows: the OF must be embedded between two layers parallel to the reinforcement fibers, and the volume fraction of the embedded OF needs to be low.

No significant additional decrease in interlaminar shear strength was observed after TVac conditioning, indicating that TVac conditioning has no further effect on the integrity of the CFRP coupons. However, it should be noted that only three coupons were available for the ILSS-REF and the ILSS-CAP (non-TVac-conditioned) series.

## 5. Conclusions

In this work, the intention was (i) to assess the impact on the structural integrity of CFRP composite coupons with embedded OFs or capillaries, used for temperature measurements, in LEO conditions, and (ii) to confirm the functionality of the embedded FBGs. The simulation of LEO conditions was achieved through thermal vacuum conditioning.

Firstly, the functionality was assessed by comparing the FBG spectra before and after TVac conditioning. No peak distortion or decrease in the intensity of the FBG reflection spectrum was observed. Additionally, a comparison between surface-mounted FBGs applied after TVac conditioning and embedded FBGs showed excellent agreement.

Secondly, the impact of embedding an OF or capillary in CFRP structures in combination with TVac conditioning was studied. Visual inspection revealed no significant difference when comparing the laminate and the embedded structures (OF and capillary) in TVac-conditioned coupons with non-conditioned coupons. Visual inspection served as an initial indication that the structural integrity is indeed unaffected by TVac conditioning. This finding was corroborated by the mechanical tests, as in general, no difference in strength was found when comparing TVac-conditioned coupons with unconditioned coupons within the same type (reference, with embedded OF, or with embedded capillary). No difference in tensile strength was found between TVac-conditioned coupons with and without embedded OFs. For the coupons with embedded capillaries, the mean compressive strength was slightly lower for non-conditioned coupons, although still within the boundaries of the standard deviation of the unconditioned coupons. A slightly higher ultimate compressive strain and interlaminar shear strength were noted for TVac-conditioned reference coupons compared to the coupons with embedded OF or capillary. However, this could be attributed to the small dimensions of the coupons, increasing the relative influence of embedded structures. In conclusion, the obtained results were in line with previous findings: When a limited number of OFs are embedded parallel to the reinforcement fibers, the strength of the CFRP coupons is unaffected. In addition, no influence on the structural integrity of the coupons caused by TVac conditioning could be identified.

The main conclusion was that small CFRP coupons with embedded OFs or capillaries successfully withstand 10 TVac cycles, and the FBG measuring functionality remains intact. With the validity of embedded FBGs established in this paper, the next steps involve utilizing them to measure and validate residual strain. Additionally, these OFs with FBGs can be employed in large-scale CFRP structures to test SHM monitoring techniques, such as damage detection, vibration monitoring, and fingerprinting. Finally, it should be noted that although in this paper FBG-based sensors were tested, the results can be extrapolated to other sensing techniques based on optical fibers made from the same material and having similar dimensions.

## Figures and Tables

**Table 1:** Overview of the lay-up and dimensions of manufactured coupons per test.

	Lay-Up	Width [mm]	Nominal Length [mm]	Thickness [mm]
Tensile test	[90/0]_3s_	25	250	2.5
Compression test	[0/90]_3s_	25	150	2.5
ILSS test	[0/90]_3s_	10	20	2.5

**Table 2:** Overview of the different samples produced and how many have been TVac conditioned. “REF” stands for reference coupons without embedded OF or capillary; “OF” and “CAP” stand for coupons with embedded OF and capillaries, respectively.

	TVac				No TVac				Total
	REF	OF	CAP	Total	REF	OF	CAP	Total	
Tensile test	5	5	-	10	-	-	-	0	10
Compression test	5	5	5	15	-	-	5	5	20
ILSS test	5	5	5	15	3	5	3	11	26

**Table 3:** The measured ultimate strain values of the embedded and surface-mounted FBG sensors.

Coupon ID	Ultimate Strain Surface-Mounted Ref Sensor [%]	Ultimate Strain Embedded Sensor [%]	Correlation Embedded Sensor/Surface-Mounted Sensor
**ST-fib-01**	1.549	1.541	0.994
**ST-fib-02**	1.253	1.264	1.008
**ST-fib-03**	1.473	1.470	0.996
**ST-fib-04**	1.402	1.406	1.001
**ST-fib-05**	1.347	1.332	1.016
**Average**	1.405	1.403	1.003
**STD**	0.102	0.098	0.008

**Figure A1:**
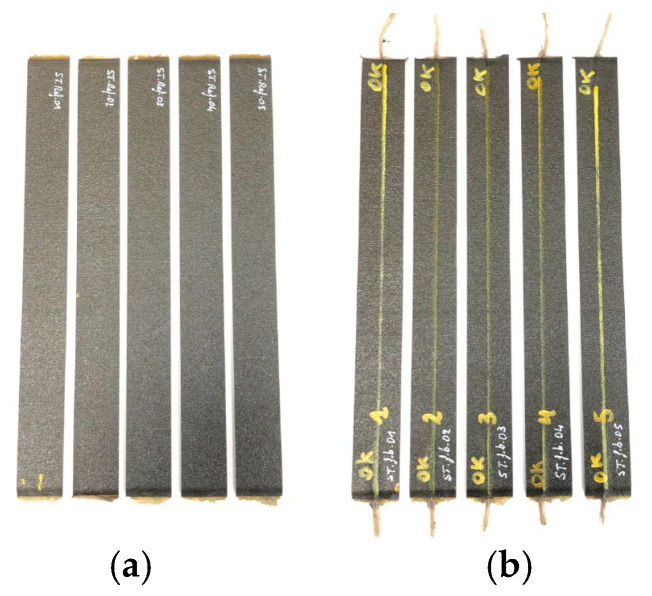
Tensile test coupons overview. (**a**) Reference coupons without embedded OF/capillary. (**b**) Coupons with 80/120 µm fiber embedded along the yellow line.

**Figure A2:**
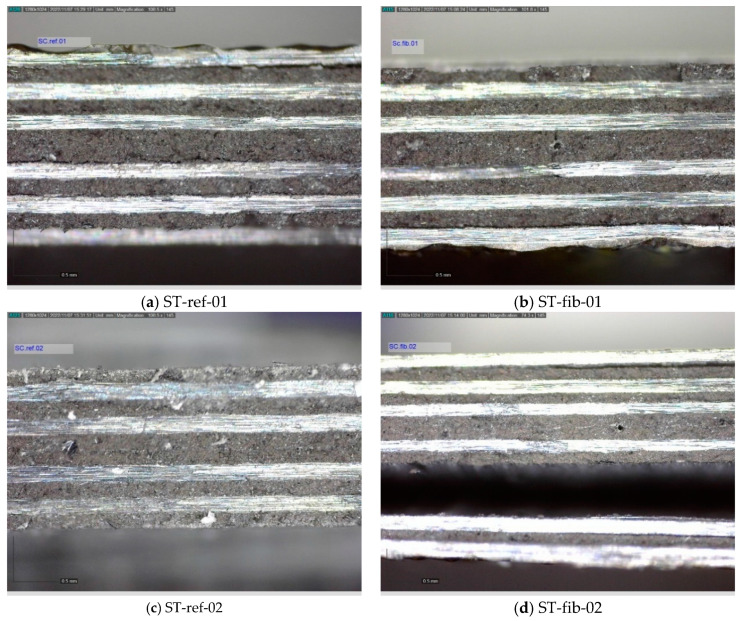

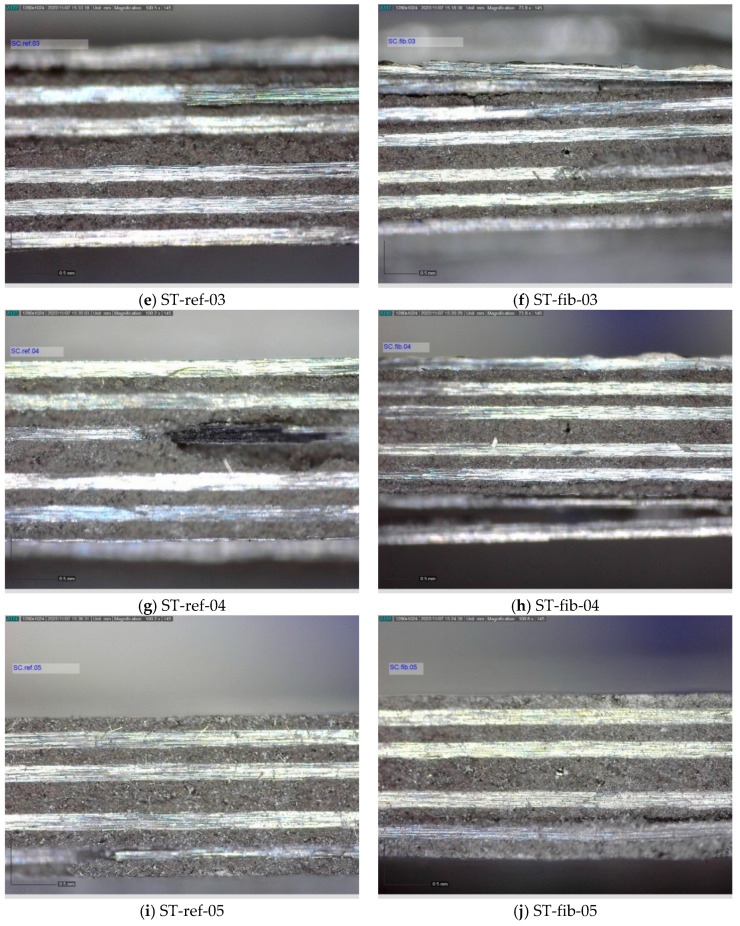
Cross-sections of the tensile test coupons taken after TVac conditioning.

**Figure A3:**
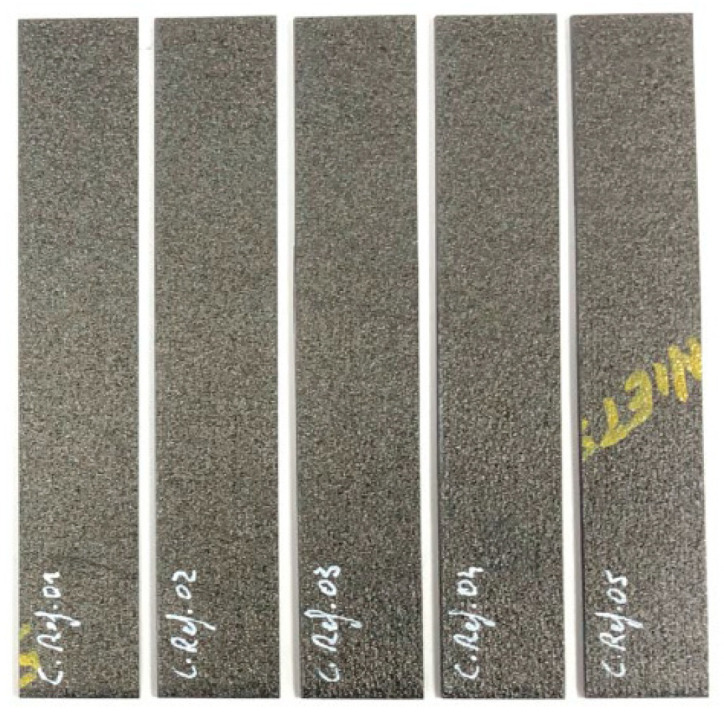
Compression test reference coupons without embedded OF/capillary.

**Figure A4:**
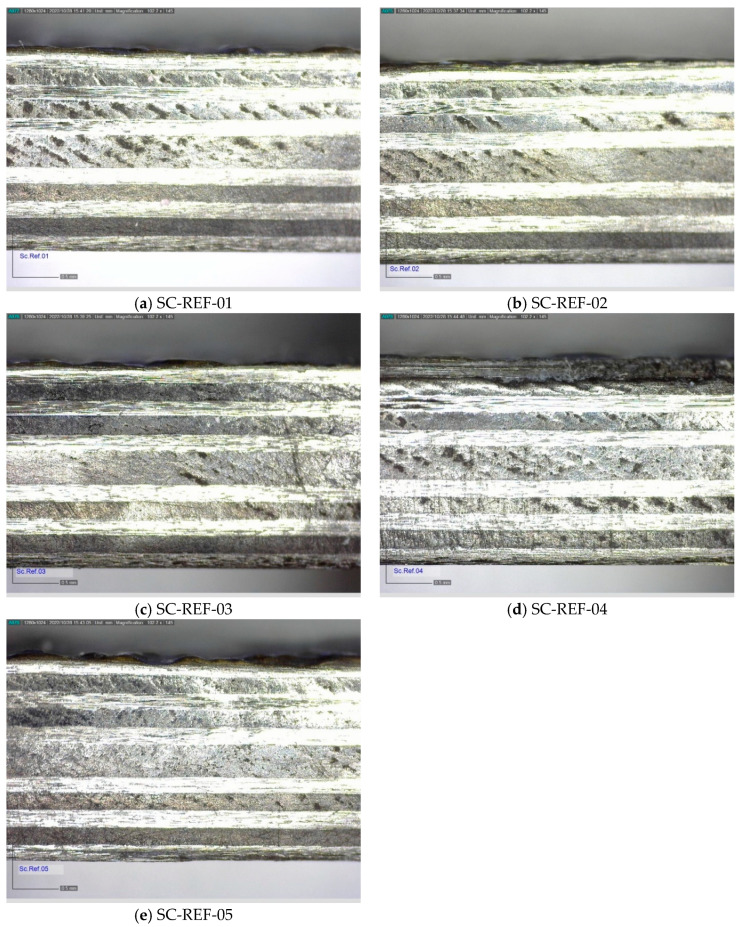
Cross-sections of the compression test reference coupons without embedded OFS/capillary taken after TVac conditioning.

**Figure A5:**
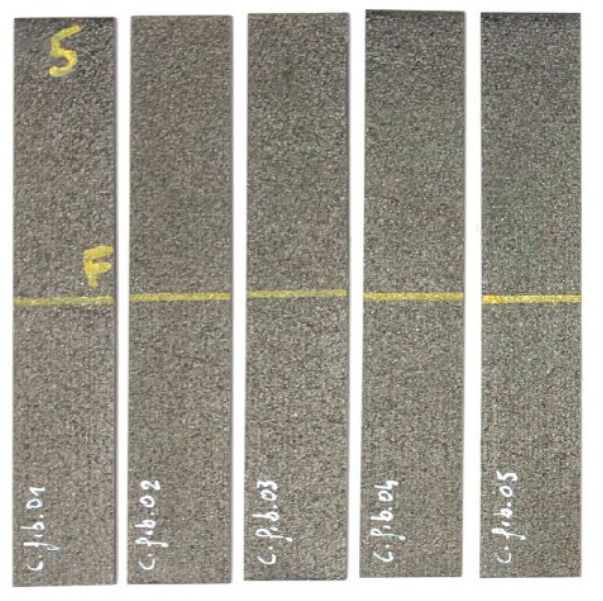
Compression test coupons with 80/120 µm fiber embedded along the yellow line.

**Figure A6:**
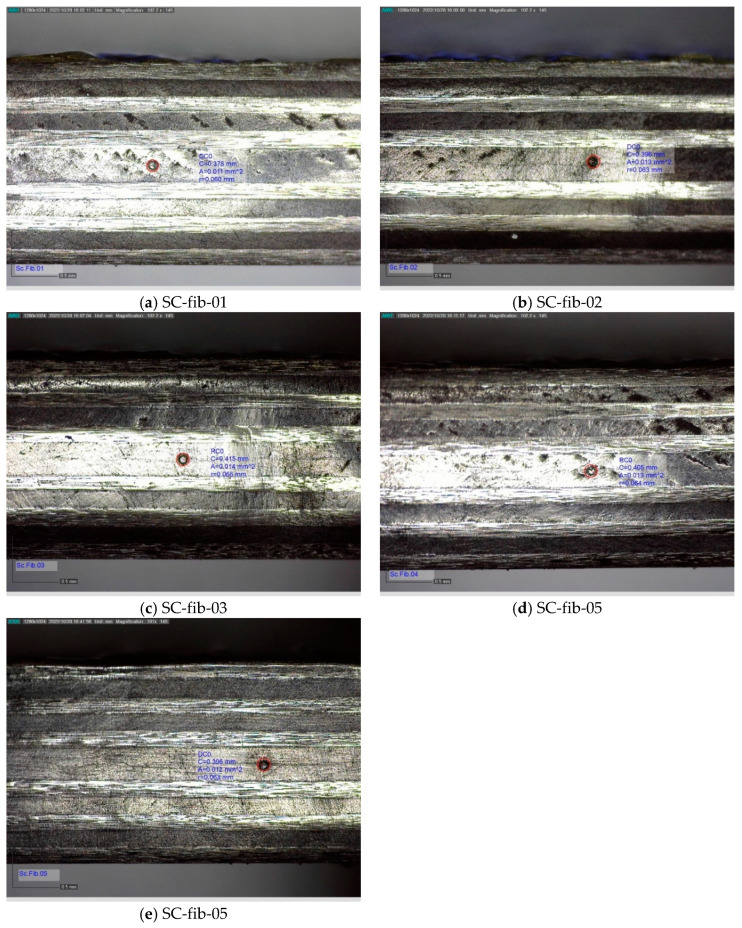
Cross-sections of the compression test coupons with embedded OF taken after TVac conditioning.

**Figure A7:**
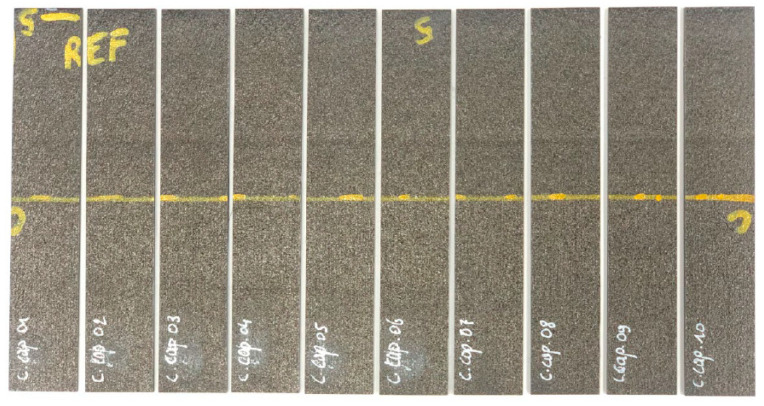
Compression test coupons with 250/360 µm capillary embedded along the yellow line.

**Figure A8:**
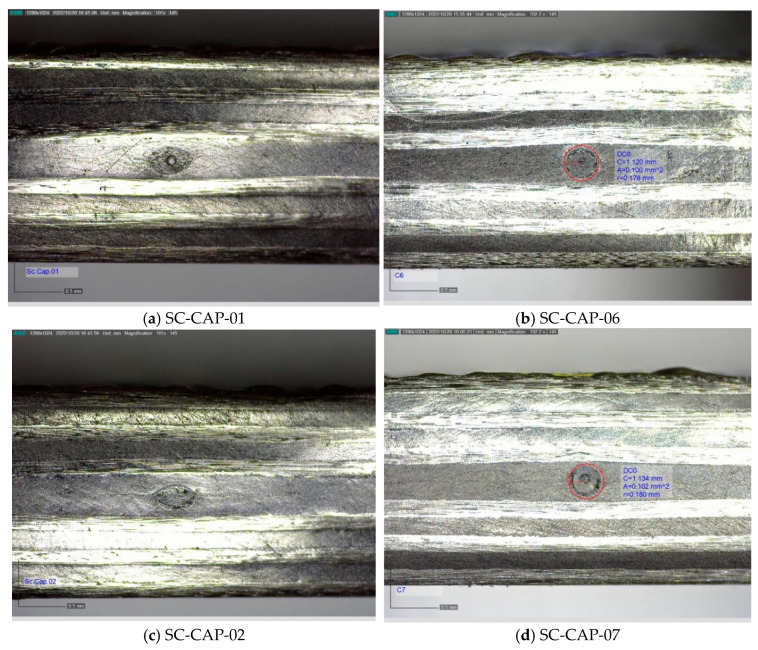

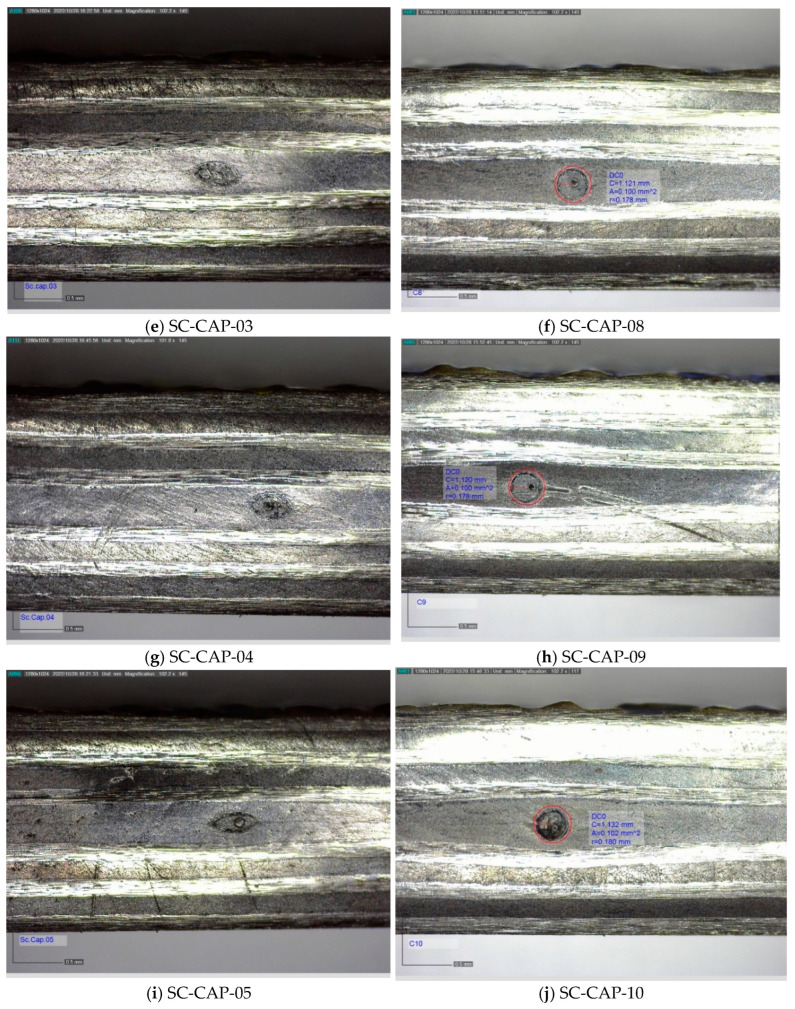
Cross-sections of the compression test coupons with embedded capillary. The figures on the (**left** (**a**,**c**,**e**,**g**,**i**)) are from the coupons that were not TVac conditioned. The figures on the (**right** (**b**,**d**,**f**,**h**,**j**)) are taken after TVac conditioning.

**Figure A9:**
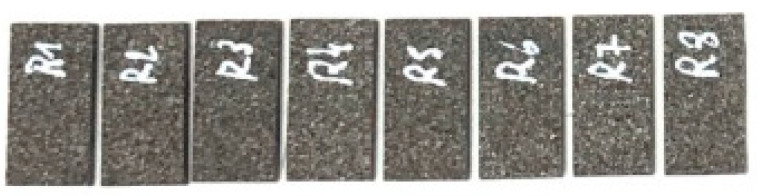
ILSS reference coupons without embedded OF/capillary.

**Figure A10:**
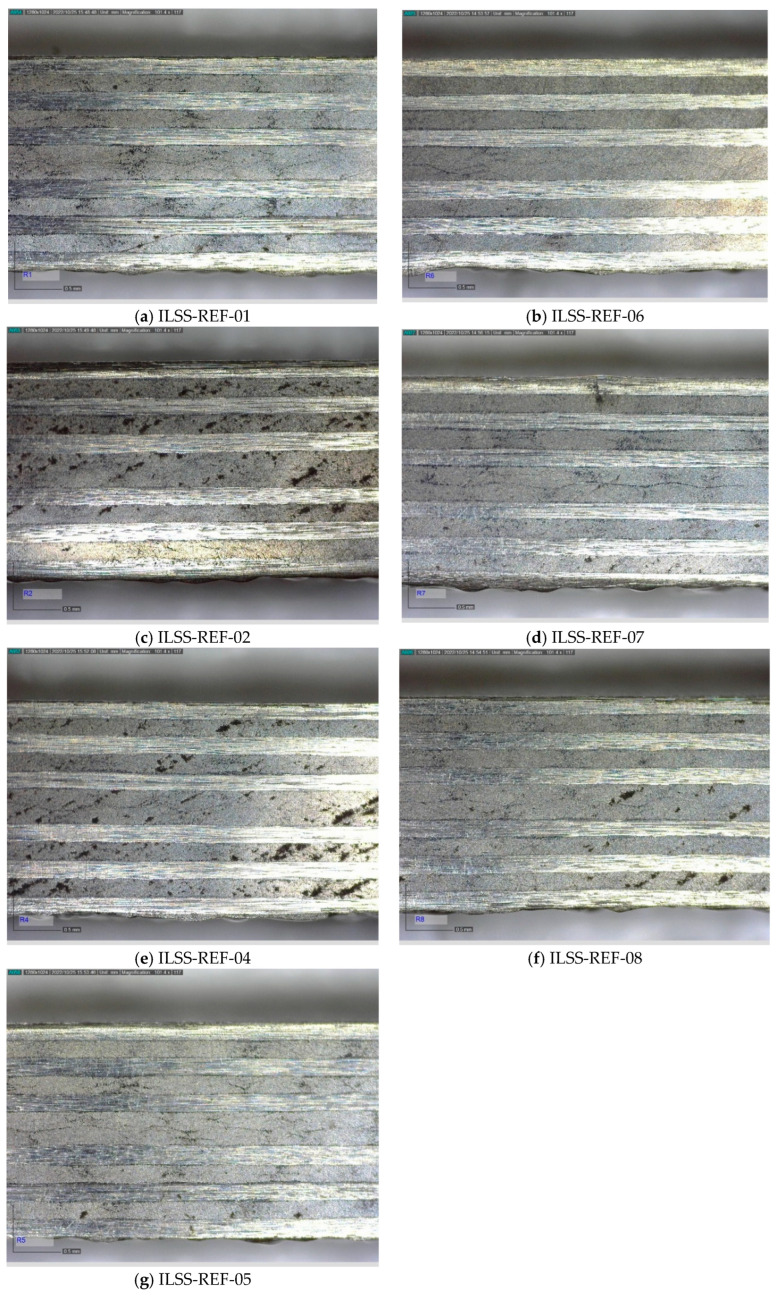
Cross-sections of the ILSS test reference coupons without embedded OF/capillary. The figures on the (**left** (**a**,**c**,**e**,**g**)) are from coupons after TVac conditioning. The figures on the (**right** (**b**,**d**,**f**)) are from coupons that were not TVac conditioned. Note that the cross-section of ILSS-REF-03 is missing.

**Figure A11:**
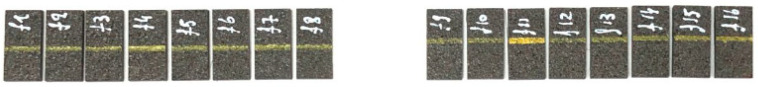
ILSS test coupons with 80/120 µm fiber embedded along the yellow line.

**Figure A12:**
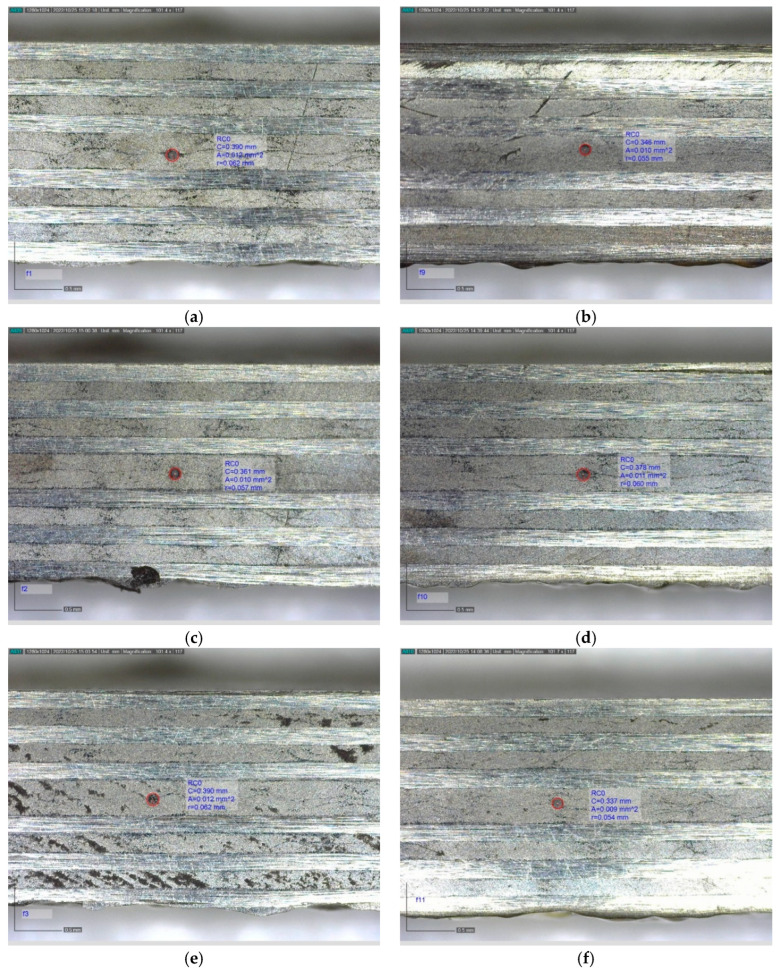

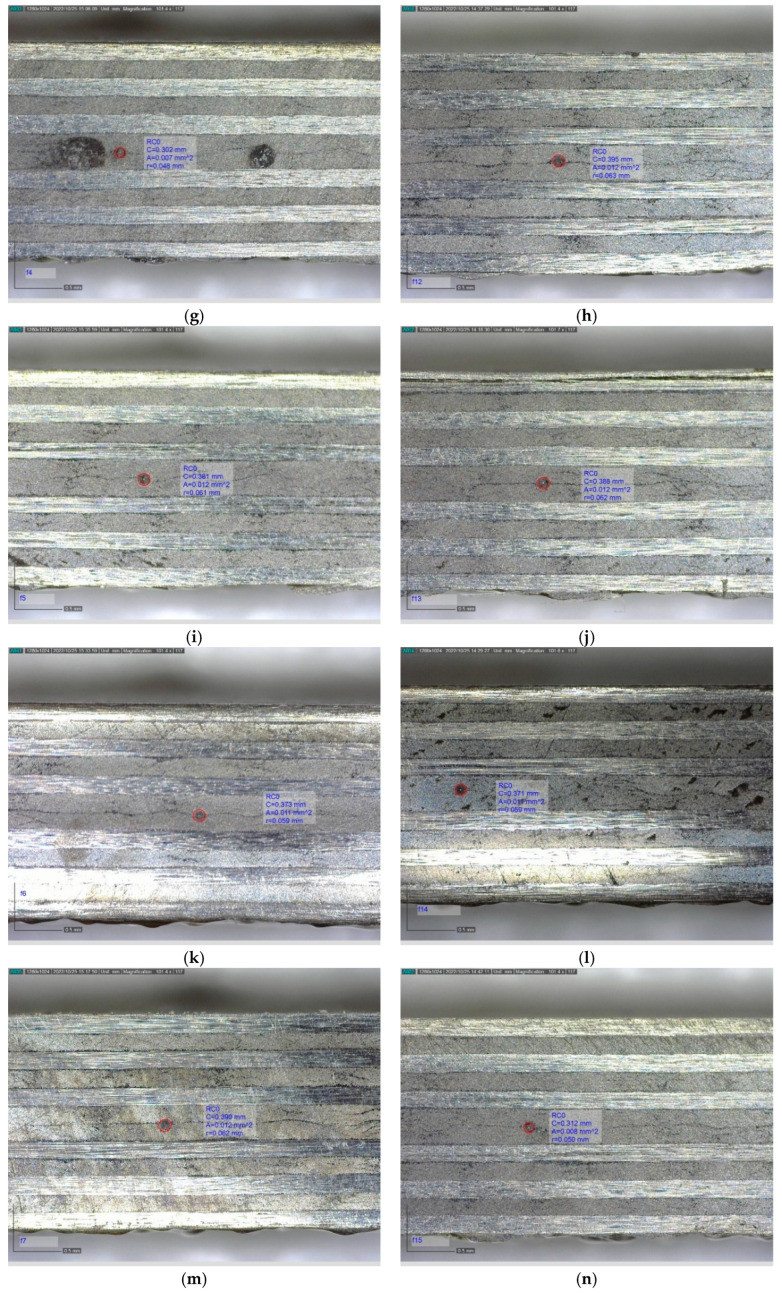

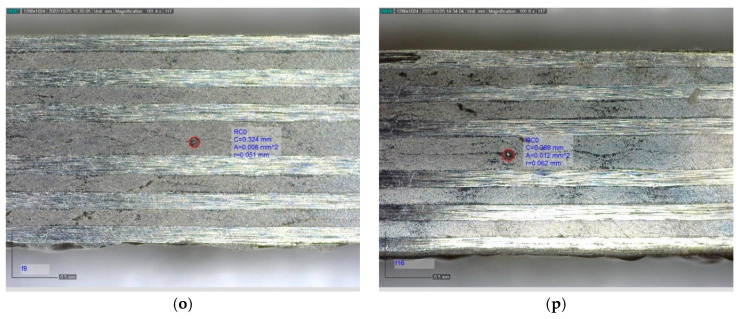
Cross-sections of the ILSS test coupons without embedded OF. The figures on the (**left** (**a**,**c**,**e**,**g**,**i**,**k**,**m**,**o**)) are from coupons after TVac conditioning. The figures on the (**right** (**b**,**d**,**f**,**h**,**j**,**l**,**n**,**p**)) are from coupons that were not TVac conditioned.

**Figure A13:**
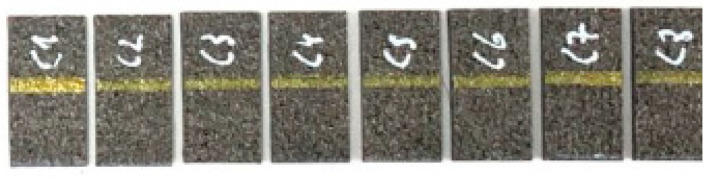
ILSS test coupons with 106/160 µm fiber embedded along the yellow line.

**Figure A14:**
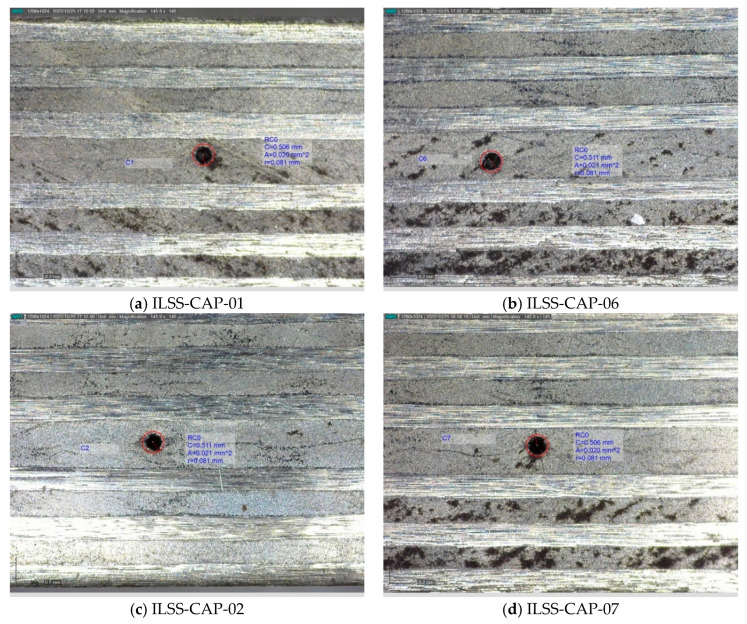

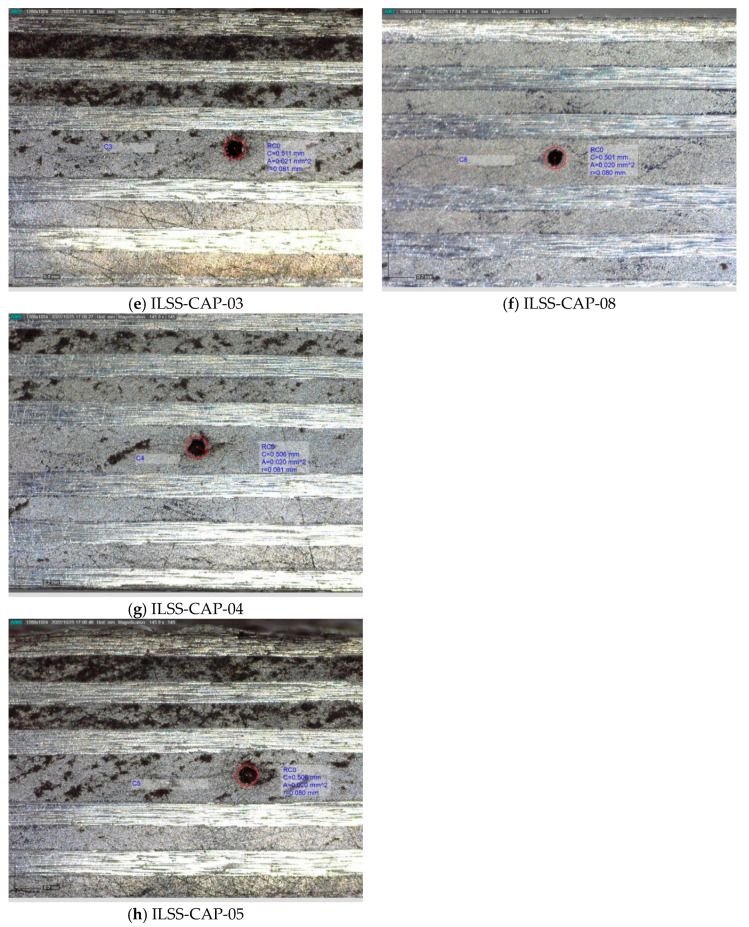
Cross-sections of the ILSS test coupons without embedded capillary. The figures on the (**left** (**a**,**c**,**e**,**g**,**h**)) are from coupons after TVac conditioning. The figures on the (**right** (**b**,**d**,**f**)) are from coupons that were not TVac conditioned.

## Data Availability

Data are contained within the article.

## Appendix B. All Test Data

### Appendix B.1. Tensile Test Data

**Table A1:** Tensile test results for the reference coupons without embedded OF with FBG.

Coupon ID	TVac	OF/ No OF	Ultimate Strain Surface-Mounted Ref Sensor [%]	Ultimate Strain Embedded [%]	Ultimate Stress [MPa]	Correlation Embedded Sensor/Surface-Mounted Sensor	R^2^	Stifness E_comp_ [GPa]	Broken Mid-Section/Tab Section
ST-Ref-01	Yes	No	1.474	-	1046	-	-	72.2	Mid-section
ST-Ref-02	Yes	No	1.413	-	977	-	-	70.4	Tab section
ST-Ref-03	Yes	No	1.398	-	980	-	-	72.4	Mid-section
ST-Ref-04	Yes	No	1.432	-	970	-	-	71.4	Mid-section
ST-Ref-05	Yes	No	1.321	-	921	-	-	71.5	Tab section
AVERAGE			1.408	-	979	-	-	71.58	
STD			0.050	-	40			0.71	
CV			3.6%	-	4.1%			1.0%	

**Table A2:** Tensile test results for the coupons with embedded OFS.

Coupon ID	TVac	OF/ No OF	Ultimate Strain Surface-Mounted Ref Sensor [%]	Ultimate Strain Embedded [%]	Ultimate Stress [MPa]	Correlation Embedded Sensor/Surface-Mounted Sensor	R²	Stifness Ecomp [GPa]	Broken Mid-Section/Tab Section
ST-fib-01	Yes	Yes	1.549	1.541	1091	0.994	1	71.5	Mid-section
ST-fib-02	Yes	Yes	1.253	1.264	885	1.008	1	72.2	Mid-section
ST-fib-03	Yes	Yes	1.473	1.470	1034	0.996	1	71.4	Mid-section
ST-fib-04	Yes	Yes	1.402	1.406	984	1.001	1	71.2	Mid-section
ST-fib-05	Yes	Yes	1.347	1.332	903	1.016	1	70.0	Mid-section
AVERAGE			1.405	1.403	979	1.003	1	71.26	
STD			0.102	0.098	78	0.008		0.71	
CV			7.2%	7.0%	7.9%	0.8%		1.0%	

### Appendix B.2. Compression Test Data

**Table A3:** Compression test results for the reference coupons without embedded OF.

Coupon ID	TVac	OF/ No OF/CAP	Ultimate Strain Surface-Mounted Ref Sensor [%]	Ultimate Stress [MPa]	Stiffness E_chord_ [GPa]	Broken Mid-Section/Tab Section
SC-Ref-01	Yes	No OF	−1.195	601	−55.7	Mid-section
SC-Ref-02	Yes	No OF	−1.380	701	−58.8	Mid-section
SC-Ref-03	Yes	No OF	−1.285	665	−58.1	Mid-section
SC-Ref-04	Yes	No OF	−1.374	694	−56.9	Mid-section
SC-Ref-05	Yes	No OF	−1.802	657	−43.0	Mid-section
AVERAGE			−1.3085	663.6	−57.4	
STD			0.076	35.460	1.2	
CV			−5.8%	5.3%	−2.1%	

**Table A4:** Compression test results for the coupons with embedded OFS.

Coupon ID	TVAC	OF/ No OF/CAP	Ultimate Strain Surface-Mounted Ref Sensor [%]	Ultimate Stress [MPa]	Stifness E_chord_ [GPa]	Broken Mid-Section/Tab Section
SC-fib-01	Yes	OF	-	638	−53	Mid-section
SC-fib-02	Yes	OF	−1.224	675	−56.4	Mid-section
SC-fib-03	Yes	OF	−1.166	650	−60.5	Mid-section
SC-fib-04	Yes	OF	−1.203	697	−57.5	Mid-section
SC-fib-05	Yes	OF	−1.303	678	−53.9	Mid-section
AVERAGE			−1.224	667.6	−56.3	
STD			0.050	21.03901	2.7	
CV			4.1%	3.2%	−4.8%	

**Table A5:** Compression test results for the coupons with embedded capillary. No TVac conditioning was applied.

Coupon ID	TVac	OF/ No OF/CAP	Ultimate Strain Surface-Mounted Ref Sensor [%]	Ultimate Stress [MPa]	Stifness E_chord_ [GPa]	Broken Mid-Section/Tab Section
SC-CAP-01	No	CAP	−1.231	683	−60.1	Mid-section
SC-CAP-02	No	CAP	−1.272	692	−56.8	Mid-section
SC-CAP-03	No	CAP	−1.299	677	−56.3	Mid-section
SC-CAP-04	No	CAP	−1.172	578	−59.5	Mid-section
SC-CAP-05	No	CAP	−1.293	672	−54.4	Mid-section
AVERAGE			−1.253	660	−57.4	
STD			0.047	47	2.1	
CV			3.8%	7.1%	3.7%	

**Table A6:** Compression test results for the coupons with embedded capillary. These coupons were TVac conditioned.

Coupon ID	TVAC	OF/ No OF/CAP	Ultimate Strain Surface-Mounted Ref Sensor [%]	Ultimate Stress [MPa]	Stifness E_chord_ [GPa]	Broken Mid-Section/Tab Section
SC-CAP-06	Yes	CAP	−1.245	657	−59.5	Mid-section
SC-CAP-07	Yes	CAP	−1.313	624	−54.3	Mid-section
SC-CAP-08	Yes	CAP	−1.254	632	−57.6	Mid-section
SC-CAP-09	Yes	CAP	−1.154	614	−57.4	Mid-section
SC-CAP-10	Yes	CAP	−1.218	629	−60.5	Mid-section
AVERAGE			−1.237	631	−57.9	
STD			0.052	14.519	2.1	
CV			−4.2%	2.3%	3.7%	

### Appendix B.3. ILSS Test

**Table A7:** ILSS test results for the reference coupons without embedded OF. The first 5 coupons were TVac conditioned, the 3 last coupons were not.

Coupon ID	TVac	OF/ No OF/CAP	Interlaminar Shear Strength [MPa]	Broken at Neutral Axis? Yes/No
ILSS-REF-01	Yes	No OF	93.9	Yes
ILSS-REF-02	Yes	No OF	72.7	Yes
ILSS-REF-03	Yes	No OF	94.4	Yes
ILSS-REF-04	Yes	No OF	91.7	Yes
ILSS-REF-05	Yes	No OF	94.1	Yes
AVERAGE			89.4	
STD			8.4	
CV			9.4%	
ILSS-REF-06	No	No OF	89.8	Yes
ILSS-REF-07	No	No OF	76.7	Yes
ILSS-REF-08	No	No OF	93.7	Yes
AVERAGE			86.7	
STD			7.3	
CV			8.4	

**Table A8:** ILSS test results for the coupons with embedded OF. The first 5 coupons were TVac conditioned, the 5 last coupons were not.

Coupon ID	TVac	OF/ No OF/CAP	Interlaminar Shear Strength [MPa]	Broken at Neutral Axis? Yes/No
ILSS-FIB-01	Yes	OF	79.64	Yes
ILSS-FIB-02	Yes	OF	84.98	Yes
ILSS-FIB-03	Yes	OF	73.15	Yes
ILSS-FIB-04	Yes	OF	80.71	Yes
ILSS-FIB-05	Yes	OF	86.89	Yes
AVERAGE			81.1	
STD			4.8	
CV			5.9%	
ILSS-FIB-06	No	OF	91.01	Yes
ILSS-FIB-07	No	OF	73.57	Yes
ILSS-FIB-08	No	OF	88.97	Yes
ILSS-FIB-09	No	OF	71.05	Yes
ILSS-FIB-10	No	OF	85.82	Yes
AVERAGE			82.1	
STD			8.2	
CV			10.0%	

**Table A9:** ILSS test results for the coupons with embedded capillary. The first 5 coupons were TVac conditioned, the 3 last coupons were not.

Coupon ID	TVac	OF/ No OF/CAP	Interlaminar Shear Strength [MPa]	Broken at Neutral Axis? Yes/No
ILSS-CAP-01	Yes	CAP	73.2	Yes
ILSS-CAP-02	Yes	CAP	79.8	Yes
ILSS-CAP-03	Yes	CAP	86.1	Yes
ILSS-CAP-04	Yes	CAP	77.4	Yes
ILSS-CAP-05	Yes	CAP	87.5	Yes
AVERAGE			80.8	
STD			5.4	
CV			6.6%	
ILSS-CAP-06	No	CAP	71.6	Yes
ILSS-CAP-07	No	CAP	83.0	Yes
ILSS-CAP-08	No	CAP	82.3	Yes
AVERAGE			79.0	
STD			5.2	
CV			6.6%	
